# Evaluation of the effect of different irrigation solutions used in regenerative endodontic treatment of necrotic molar teeth with open apex on postoperative pain- randomized clinical trial

**DOI:** 10.1007/s00784-025-06153-3

**Published:** 2025-01-16

**Authors:** İpek Eraslan Akyüz, Ayşe Tuğba Eminsoy Avcı, Yakup Üstün, Kevser Solak Kolçakoğlu, Esra Kızılcı

**Affiliations:** 1 Department of Endodontics, Faculty of Dentistry, Erciyes University, Melikgazi, Kayseri, 38039 Türkiye; 2 Department of Pedodontics, Faculty of Dentistry, Erciyes University, 38039 Kayseri, Türkiye

**Keywords:** Apical periodontitis, Devitalized teeth, Post-operative pain, Regeneration

## Abstract

**Objectives:**

This study evaluates the effect of different irrigation solutions for postoperative pain in the regenerative endodontic treatments (RET) of necrotic teeth with open apex.

**Materials and methods:**

This study included necrotic, deeply carious lower molars of 42 patients. Access cavities of the teeth were opened and working lengths were measured at the first visit. In Group 1, the root canals were irrigated with 1.5% sodium hypochlorite (NaOCl) and 17% Ethylenediaminetetraacetic-acid (EDTA), in Group 2, with 1.5% NaOCl and 10% citric acid, and in the Group 3, with 1.5% NaOCl and 9% Etidronic acid (HEDP) mixture solution. Final irrigation was performed with distilled water. The canals were dried, filled with calcium hydroxide paste, and sealed with Cavit. Postoperative pain was assessed using a numerical rating scale, and Paracetamol was prescribed. At the second visit, calcium hydroxide was removed with 1.5% NaOCl, irrigated with chelators, and sealed with Sure-Seal PT 3 mm below the cementoenamel junction before composite resin filling.

**Results:**

Postoperative pain was higher in Group 3 (*p* < 0.05), but analgesic consumption did not show a significant difference (*p* > 0.05).

**Conclusions:**

HEDP caused significantly more postoperative pain than EDTA and citric acid (*p* < 0.05). While the study provided information regarding the effects of irrigation solutions on postoperative pain, further research is needed as it did not include detailed assessments of long-term periapical healing and apex closure.

**Clinical relevance:**

When HEDP is used as a chelation agent in RETs, it shows a higher degree of postoperative pain than EDTA and citric acid solutions.

**Clinical trial registration:**

The study protocol was registered at www.clinicaltrials.gov (ID: NCT 06386991).

## Introduction

Caries, traumatic dental injuries, and dental anomalies can damage the pulp-dentin complex of teeth. This may cause pulp necrosis and disruption in the development process of teeth [1]. Root canal treatment for mature permanent teeth with necrotic pulp has a long-term success rate of over 86% [2]. However, the sensitive root physiology and anatomy of teeth with immature apexes lead to difficulties in performing root canal treatment and more complex diagnoses [3]. Regenerative endodontic treatment (RET) saves immature teeth with necrotic pulp. RET is a biological procedure designed to repair or replace damaged structures, including dentin, root structures, and pulp-dentin complex cells. Continuous root development, including apical closure and root thickening, is possible with this treatment.

Most today used irrigants are chemically active solutions, and their direct reaction with biofilm is considered the foundation of their antimicrobial effect [4]. However, irrigants also interact with other substrates within the root canal system, such as dentin or other irritants [5]. In regenerative endodontic treatments, the goal is to create an environment that supports root maturation, though no standardized irrigation protocol exists. Despite this, high success rates and favorable treatment outcomes have been reported [6, 7]. In the current clinical procedures of the American Association of Endodontists, irrigation with 17% ethylenediaminetetraacetic acid (EDTA) is recommended after the use of low-concentration sodium hypochlorite (NaOCl) [8]. EDTA minimizes the cytotoxicity of NaOCl and increases the release of bioactive molecules in the dentin matrix [9]. Moreover, NaOCl is unable to dissolve inorganic components of the smear layer. Acids are usually used to remove inorganic components. EDTA is one of the most popular irrigation solutions among these acid solutions. Calcium complexing (chelating) agents such as EDTA are thus recommended as additional agents in root canal treatment, either in liquid form or in paste-type preparations [10]. Some other acid solutions have a similar effect to the EDTA solution, such as Citric acid and etidronic acid (HEDP). Citric acid is an irrigation solution that can be used as an alternative to EDTA due to its high antibacterial activity and low toxicity [11]. Concentrations between 10 and 50% are used in endodontic treatment procedures [12].

Additionally, studies examining the effects of various irrigation solutions on growth factors and stem cells found that the release of TGF-β1 from the dentin extracellular matrix was higher in 10% citric acid than in 17% EDTA [13]. Another acid solution that, when used alone, has sufficient effect to remove the inorganic components of the smear layer is HEDP [14]. HEDP was stated to be a weak chelator that does not harm the mineral content of dentine and does not diminish the activity of NaOCl in combined usage. Also, HEDP dissolves the smear layer when combined with NaOCl [15]. A study examining the effects of various natural and artificial chelation agents on growth factor release from dentinal tubules shows that 9% HEDP causes a higher rate of growth factor release than 17% EDTA and 1% phytic acid [16].

Calcium silicate (Ca-Si) based materials play an important role in in endodontic procedures involving pulpal regeneration and hard tissue repair, such as pulp capping, pulpotomy, apexogenesis, apexification, perforation repair, and root-end filling due to their biocompatibility [17]. Additionally, Ca-Si materials have been shown to have an effective sealing ability, which is associated with their expansion, bioactivity, ion release function as epigenetic signals, and beneficial biological properties [18]. Ca-Si materials can be categorized by their composition and formulation: powder liquid, paste-to-paste, or premixed ‘ready to use’ materials [19]. However, new products containing fast hardening and pre-mixed Calcium silicate (Ca-Si) Ca-Si have been introduced due to difficulty mixing and late hardening time. All premixed Ca-Si materials are characterized by different chemical compositions of radiopacifiers and thickening agents and possess varying percentages of Ca-Si (the bioactive component) [17]. Sure-Seal Root PT (Sure Dent Corporation, Gyeonggi-do, South Korea) is a Ca-Si-based paste that is pre-mixed and ready to apply. Ca-Si ingredient components play a positive role in the activation of undifferentiated stem cells [20]. The high pH of Ca-Si materials may also provide several biological advantages, such as promoting hard tissue formation and interfering with osteoclastic activity, leading to favorable healing [21].

There are many studies in which different regenerative endodontic treatment procedures are applied, and follow-up results are reported [22–24]. Ahmed et al. [25] compared the effects of different regenerative endodontic procedures on postoperative pain. To our knowledge, no study in the literature evaluates the effect of varying irrigation solutions on postoperative pain in teeth undergoing regenerative endodontic treatment.

This study aims to evaluate the effect of using different irrigation solutions on postoperative pain in mandibular molar teeth undergoing regenerative endodontic treatment. The null hypotheses of this study are as follows: (1) There is no difference in the effect of using different irrigation solutions in regenerative endodontic treatments on postoperative pain levels. (2) There is no difference in the effect of using different irrigation solutions in regenerative endodontic treatments on the consumption of analgesic tablets.

**Fig. 1:**
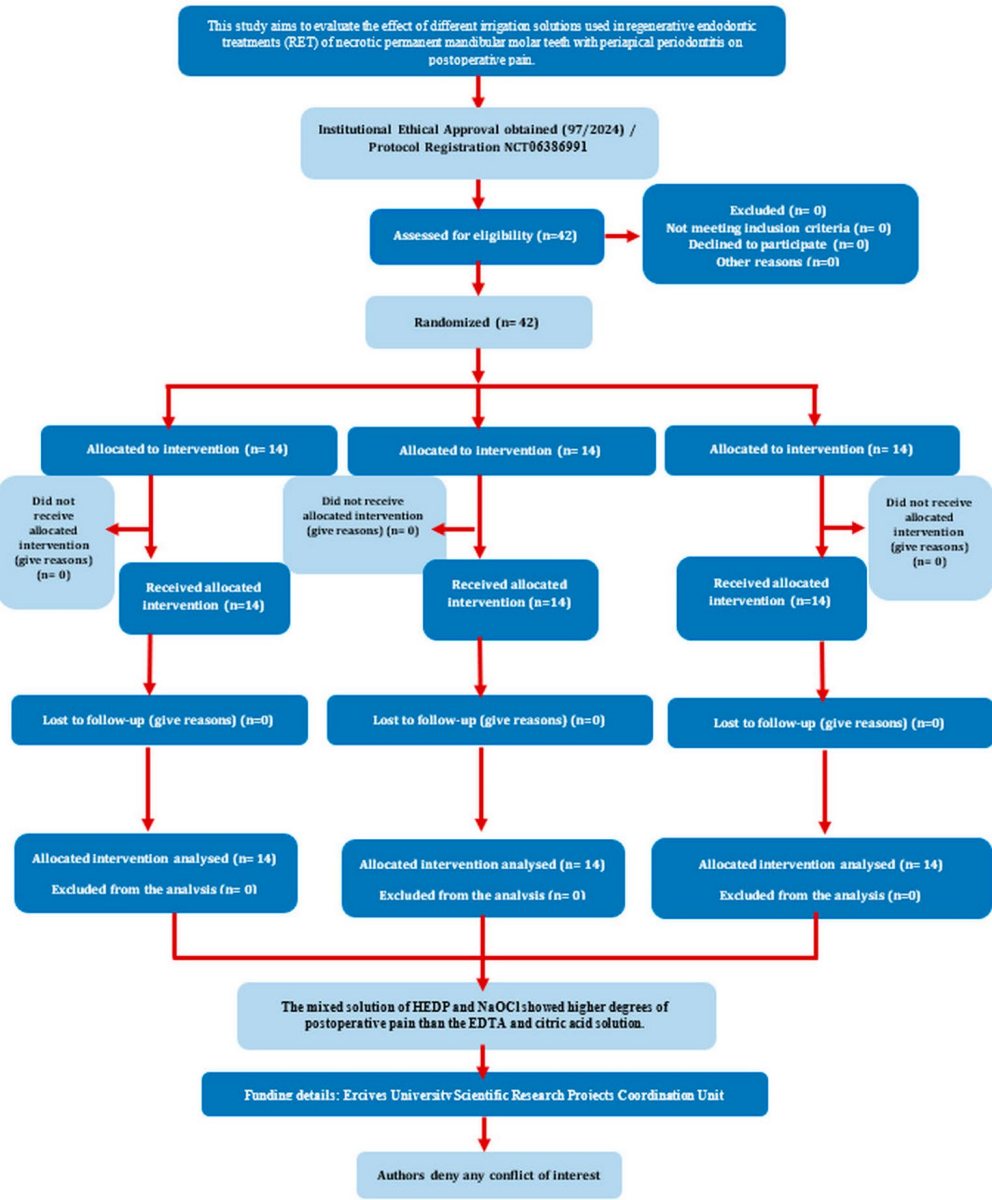
PRIRATE 2020 flowchart. *From Nagendrababu V, Duncan HF, Bjørndal L, Kvist T, Priya E, Jayaraman J, Pulikkotil SJ, Pigg M, Rechenberg DK, Vaeth M, Dummer P. (2020) PRIRATE 2020 guidelines for reporting randomized trials in Endodontics: a consensus-based development. International Endodontic Journal Mar 20. doi: 10.1111/iej.13294. For further details, visit: http://pride-endodonticguidelines.org/prirate/

## Materials and methods

### Study design and registration

This randomized clinical trial has been written according to preferred reporting items for randomized trials in endodontics (PRIRATE) 2020 guidelines [26]. The steps of this study are presented in the PRIRATE flowchart (Fig. 1). The study was designed as randomized clinical trial, and its protocol was registered at www.clinicaltrials.gov (ID: NCT 06386991). This study was approved by the institutional review board/ethical committee (approval number 2024/97). Treatment and follow-up of the study participants were done between February 2024 and June 2024. The patients were treated according to the declaration of Helsinki and as revised in 2013. The treatment process, side effects, and other treatment alternatives were explained to the patients, and informed consent was obtained with the signature of their legal representative.

### Sample size calculation

Turcosa (Turcosa Analytics Ltd.Co., Türkiye) was used for statistical analysis in this study. The sample size calculation was based on a previous study [25], using the calculated effect size of 1.39, a type I error of 0.05, and a power of 0.9, a minimum sample of 39 subjects (13 subjects per group) is required to detect a significant difference between the study groups. In the current study, 14 patients were identified for each group.

### Randomization

The method used was simple randomization. A random sequence of numbers (1–42) was generated by a co-investigator using computer software (http://www.random.org/). The names of the chelation agents in the groups were written on papers of the same size. Forty-two pieces of paper were folded invisibly and placed in a bag. A sheet of paper was randomly selected and given to patients by an independent person not otherwise involved in the study. To maintain the integrity of the randomization, he had no access to patient information or the study protocol. The participants were allocated to their respective treatment groups by a designated study coordinator who had access to the random allocation. The coordinator ensured that the allocation was concealed and that each patient was assigned to a group in a manner consistent with randomization. This assignment was performed after patients had been enrolled and baseline assessments had been completed, guaranteeing that neither patients nor investigators were aware of their group assignments during the enrolment process.

### Eligibility criteria

The included patients were healthy (Category: American Society of Anesthesiologists class 1). This study included the necrotic, deeply carious mandibular molars of patients born between 2009 and 2015. Preoperative periapical films of the relevant teeth were evaluated, and teeth determined as Stages 2 and 3 according to the Cvek classification were included in the current study [27]. Patients were excluded if they had a systemic disease or were on systemic corticosteroid therapy, patients reported bruxism or clenching, took analgesics or other drugs that might alter their pain during the last 12 h preoperatively, or had a history of allergic reactions to any of the medications or materials used. Patients with a tooth with severely curved root canals, vertical root fracture, coronal perforation, calcification or more significant than Grade I mobility, or pocket depth greater than 5 mm were also excluded.

### Diagnosis and treatment procedure

A comprehensive explanation was given to the patient and their parents about the treatment procedures, side effects, and treatment alternatives, and informed consent was obtained. Patients with pain from mandibular molar teeth with open apexes, with or without periapical radiolucency, were included in this study. The gender of these patients was 22 male and 20 female. Gender distribution in the groups was divided into an EDTA group (Male: 7, Female: 7), a citric acid group (Male: 8, Female: 6), and an HEDP group (Male: 7, Female: 7). Before starting treatment, patients were asked to record their preoperative pain level using the numerical rating scale (NRS) chart and provide a number between 0 and 100 for pain intensity. Patients with preoperative dental pain who pointed between 70 and 100 scores in NRS were included in the study. Teeth were evaluated clinically and radiographically. The patients’ teeth with severe pain on percussion and giving a negative response to the electric pulp vitality test (Parkell, Inc, Edgewood, NY) and cold test were included in the present study.

Two calibrated experienced endodontists performed all the procedures. Endodontists were blinded concerning the patients. Regenerative endodontic treatment is administered to patients in two visits. Mandibular anesthesia (2% lidocaine with 1:100,000 epinephrine) was performed. After completely removing caries or coronal restorations, the access cavity was opened using a round bur (Dentsply Sirona), and tooth isolation was performed using the rubber dam. Scouting, negotiation, and patency of the canals were done using stainless steel hand K-files size # 10 and #15 (Dentsply Sirona, Switzerland), followed by the determination of the working length using an electronic apex locator (Propex Pixi Apex Locator, Dentsply Sirona, Switzerland) and then confirmed with intraoral periapical radiograph to be 0.5–1 mm shorter than the radiographic apex. Minimal mechanical instrumentation was employed to effectively remove adherent biofilms and necrotic pulp tissue from the root canal system while preserving the integrity of the original canal structure. Gentle debridement was performed using K-files (sizes 15 and 20) with a controlled push-and-pull motion, ensuring thorough cleaning without compromising the canal morphology [28].

Residual necrotic pulp tissue was meticulously eliminated from the root canals through copious irrigation, ensuring thorough cleaning of the canal system. In the first group of patients with root canals, the canals were irrigated with 1.5% NaOCl, and then 17% EDTA (20 ml, 1 min.) using a 30 gauge diameter side-vented needle placed 1 mm short from the working length. The citric acid (10%) and etidronic acid (18%) concentrations were prepared by a qualified chemist, following standardized protocols to ensure consistency and accuracy for each irrigation procedure. In the second group, root canals were irrigated with 1.5% sodium hypochlorite (NaOCl) followed by 10% citric acid (20 ml, 1 min.). In groups 1 and 2, intermittent irrigation with distilled water was performed to prevent the precipitate formation from NaOCl and chelating agent interaction. In the third group of patients, an equal amount of 18% HEDP and 3% NaOCl was mixed to obtain a 9% HEDP + 1.5% NaOCl solution, and then the root canals were irrigated with this mixed solution (20 ml, 1 min.). Then, for irrigation agitation, Ultra X (Changzhou Sifary Medical Technology Co.Ltd., Changzhou, China) ultrasonic device was used (1 min.), and final irrigation was performed with distilled water (10 ml). The canals were dried with paper points (Pearl Dent CO., Vietnam). Then, the calcium hydroxide paste (UltraCal XS; Ultradent, South Jordan, UT) was placed in the canals using a lentulo spiral adjusted 2 mm shorter than the working length. A cotton pellet was put into the pulp chamber, and then the access cavity was sealed using Cavit (3 M ESPE AG, Seefeld, Germany).

### Postoperative pain assessment

At the end of the first visit, patients were educated to record their postoperative pain level by assigning a number between 0 and 100 using the numerical rating scale (NRS) chart. They were categorized into four: 0 = no pain, 10–30 = mild pain, 40–60 = moderate pain, and 70–100 = severe pain. The patients were asked to record their pain level preoperatively, at 6, 12, and 24 h, and daily for up to 7 days, and return it to the investigators at a specific time.

### Consumption of analgesic tablets

Paracetamol was prescribed in case of persistent pain. Patients were asked to write down the amount of analgesic usage on the form in which they recorded their pain levels during the seven days between the first and second visit. These were divided into three categories: 0 = no analgesic usage, 1 = one analgesic usage, and 2 = two analgesic usage. Patients were asked to record daily analgesic usage at 6, 12, and 24 h after the first visit and for up to seven days. The dose of analgesics was adjusted according to the age and weight of the patients. Information was given that antibiotics should not be taken and the operator should be contacted in case of flare-ups (severe pain and swelling).

### Second visit

**Fig. 2:**
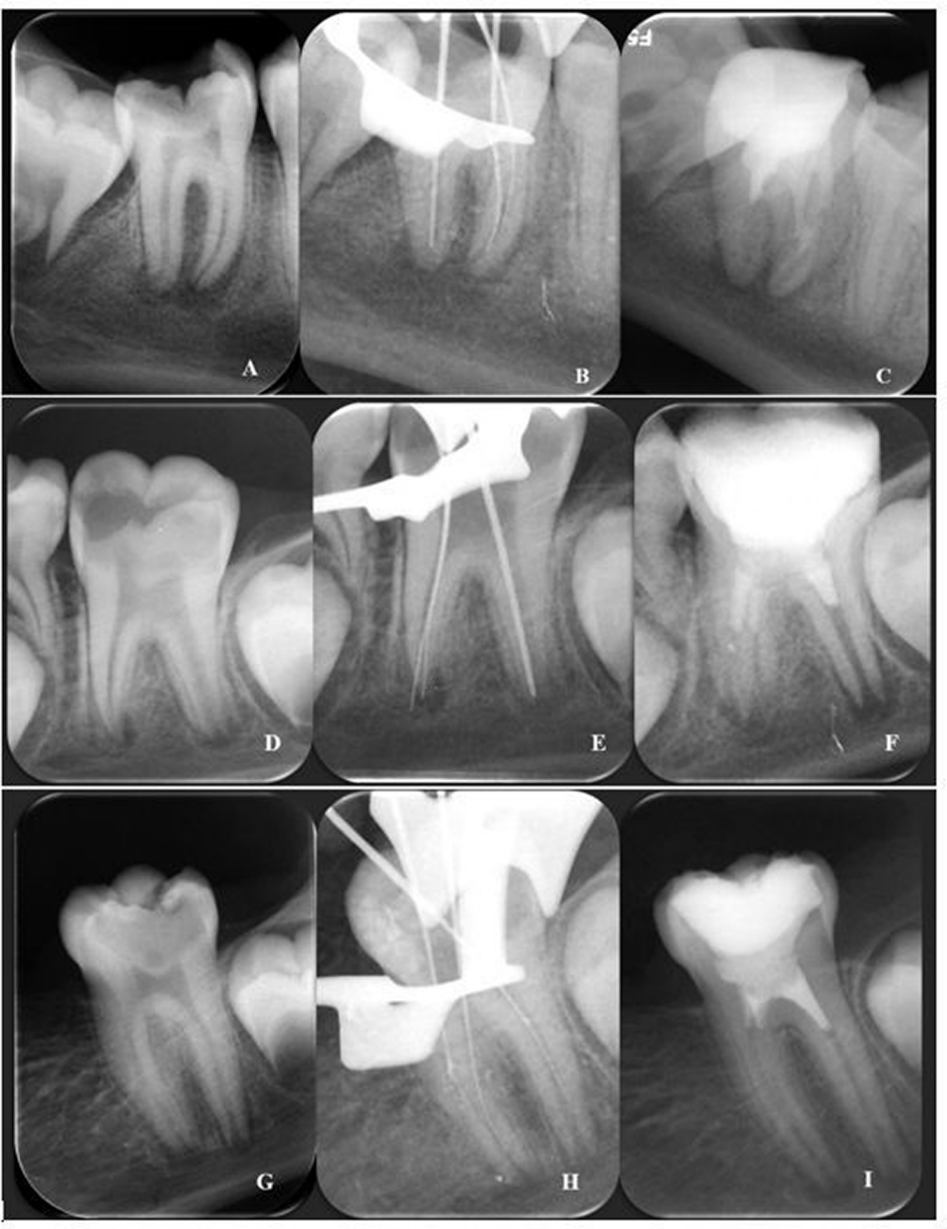
Periapical radiographs of patients undergoing regenerative treatment. (**A**) EDTA Group, initial film; (**B**) EDTA Group, working length measurement; (**C**) EDTA Group, final film. (**D**) Citric acid Group, initial film; (**E**) Citric acid Group, working length measurement; (**F**) Citric acid Group, final film. (**G**) HEDP Group, initial film; (**H**) HEDP Group, working length measurement; (**I**) HEDP Group, final film

The second visit was scheduled two weeks later. Local anesthesia of the inferior alveolar nerve block was administered with 2 mL of sefacaine (3% mepivacaine HCL, without epinephrine), followed by rubber dam application before temporary filling removal. After cleaning the temporary filling material, the calcium hydroxide paste was removed with abundant irrigation using 1,5% NaOCl (5 ml). Finally, 17% EDTA (20 ml) was used in Group 1, 10% citric acid (20 ml) was used in Group 2, and 9% etidronic acid + 1,5% NaOCl mixture solution was used in Group 3. Then, an Ultra X (Changzhou, China) ultrasonic device was used for irrigation agitation, and the canals were dried using paper points. Intentional over-instrumentation was performed with a K-file size # 10 and #15 (Dentsply Sirona, Switzerland) to induce bleeding in the apical foramen region, extending 2–3 mm beyond the apex. The file was pushed and pulled counterclockwise, and root canals were filled with blood [29]. After a blood clot formed approximately 3 mm below the cementoenamel junction, Sure Seal Root PT (Sure Dent Corporation, Gyeonggi-do, South Korea) was carefully placed over it. After the hardening period of Mineral Trioxide Aggregate (MTA) was completed, the teeth were restored with nanohybrid resin composite (Tokuyama Estelite Sigma Quick, Tokuyama, Japan) It is important to take radiography from each patient for accurate diagnosis, treatment planning, and final control. In the current study, periapical radiography was taken from all patients for preoperative, postoperative, and working length measurements (Fig. 2).

Since bleeding in the periapical region could not be achieved in the teeth of two patients, RET with PRP was performed in these patients. Ten milliliters of whole blood was drawn by venipuncture from the antecubital vein of the patient’s right arm. Blood was collected in a 15 ml sterile glass tube coated with an anticoagulant (acid-citrate dextrose). Whole blood was centrifuged (250 g for 10 min) to separate PRP and platelet-poor plasma (PPP) portions from the red blood cell fraction. PRP and PPP portions were again centrifuged (400 g for 15 min) to separate the PRP from the PPP. It was then injected into the canal space. The subsequent procedures are the same as performing RET by inducing bleeding in the periapical region.

### Statistical analysis

This study was evaluated using the IBM SPSS Statistics 21 (SPSS Inc., Chicago, IL, USA) package program. Frequency, percentage, mean, standard deviation, median, and interquartile range (IQR) were used as descriptive statistical methods in evaluating the data. Whether the data showed normal distribution was tested with the Kolmogorov-Smirnov Test. The Kruskal-Wallis H Test was applied to test the difference between variables and the difference between groups. The relationship between variables was analyzed using Spearman’s Rho. Findings were evaluated at a 95% confidence interval and 5% significance level.

**Table 1:** Analyzes of pain assessment after the first visit of regenerative endodontic treatment

Pain Assessment	*N*	Average (Standard deviation)	Median (IQR)	Mean Rank	Test Statistics	*p* Value	Pairwise Comparisons
6th Hour							
1	14	19,64(26,20)	10,00(33,00)	18,93	9,76	0,008	1–3 0,002, 2–3 0,011
2	14	19,33(34,12)	0,00(20,00)	17,07			
3	14	53,08(34,25)	50,00(60,00)	30,36			
12th Hour							
1	14	9,29(12,85)	5,00(13,00)	15,46	13,07	0,001	1–3 0,002, 2–3 0,023
2	14	19,33(40,00)	10,00(40,00)	19,30			
3	14	50,00(27,69)	50,00(30,00)	31,43			
24th Hour							
1	14	1,43(3,63)	0,00(0,00)	14,07	17,03	0,000	1–3 0,000, 2–3 0,022
2	14	13,33(22,89)	0,00(10,00)	20,27			
3	14	36,92(33,01)	30,00(45,00)	31,79			
48th Hour							
1	14	0,71(2,67)	0,00(0,00)	14,18	11,79	0,003	1–3 0,002
2	14	16,00(27,98)	0,00(20,00)	23,17			
3	14	20,77(23,62)	10,00(45,00)	28,57			
72nd Hour							
1	14	7,14(18,16)	0,00(0,00)	19,07	2,02	0,364	
2	14	10,00(18,52	0,00(10,00)	22,40			
3	14	14,62(22,22)	0,00(30,00)	24,50			
96th Hour					5,72	0,057	
1	14	-	-	18,00			
2	14	10,67(22,19)	0,00(0,00)	22,30			
3	14	21,54(32,88)	0,00(50,00)	25,68			
120th Hour					3,18	0,204	
1	14	2,86(10,69)	0,00(10,69)	18,68			
2	14	5,33(13,02)	0,00(10,00)	22,40			
3	14	12,31(18,33)	0,00(30,00)	24,89			
144th Hour					4,62	0,099	
1	14	-	-	18,00			
2	14	6,67(18,39)	0,00(0,00)	22,20			
3	14	8,46(15,73)	0,00(15,00)	24,46			
168th Hour					3,82	0,148	
1	14	-	-	20,00			
2	14	6,00(23,24)	0,00(0,00)	21,47			
3	14	10,77(27,83)	0,00(10,00)	24,57			

## Results

### Pain assessment

The Kruskal-Wallis test was used to analyze differences in postoperative pain levels across groups at various time points following the first visit of RET **(**Table 1**).** A statistically significant difference was observed between groups at the 6th, 12th, 24th, and 48th hours (*p* < 0.05). Pairwise comparisons revealed that postoperative pain levels were significantly higher in Group 3 compared to Groups 1 and 2 at the 6th, 12th, and 24th hours (*p* < 0.05). At the 48th hour, Group 3 exhibited higher pain levels than Group 1 (*p* < 0.05).

### Demographic analysis

No significant differences were found between the groups in terms of gender and age distribution (*p* > 0.05).

### Analgesic consumption

There were no statistically significant differences between the groups regarding the consumption of analgesic tablets during the postoperative period (*p* > 0.05) (Table 2).

**Table 2:** Analysis of the consumption of analgesic tablets after the first visit of regenerative endodontic treatment

The Consumption of Analgesic Tablets	*N*	Average (Standard Deviation)	Median (IQR)	Mean Rank	Test Statistics	*p* Value
6th Hour					2,65	0,266
1	14	0,29(0,47)	0,00(1,00)	21,21		
2	14	0,20(0,41)	0,00(0,00)	19,50		
3	14	0,64(0,84)	0,00(1,00)	25,46		
12th Hour					3,79	0,150
1	14	0,07(0,27)	0,00(0,00)	18,43		
2	14	0,27(0,46)	0,00(1,00)	22,33		
3	14	0,57(0,85)	0,00(1,00)	25,21		
24th Hour					1,410	0,494
1	14	0,07(0,27)	0,00(0,00)	20,46		
2	14	0,13(0,35)	0,00(0,00)	21,73		
3	14	0,36(0,75)	0,00(0,00)	23,82		
48th Hour					3,186	0,203
1	14	-	-	19,50		
2	14	0,13(0,35)	0,00(0,00)	22,30		
3	14	0,29(0,61)	0,00(0,00)	24,18		
72nd Hour					2,153	0,341
1	14	-	-	20,50		
2	14	0,07(0,26)	0,00(0,00)	21,93		
3	14	0,14(0,36)	0,29(0,73)	23,57		
96th Hour					4,24	0,120
1	14	-	-	21,00		
2	14	-	-	21,00		
3	14	0,29(0,00)	0,00(0,00)	24,07		
120th Hour					2,07	0,355
1	14	-	-	21,50		
2	14	-	-	21,50		
3	14	0,07(0,27)	0,00(0,00)	23,04		
144th Hour					2,07	0,355
1	14	-	-	21,50		
2	14	-	-	21,50		
3	14	0,14(0,54)	0,00(0,00)	23,04		
168th Hour					-	-
1	-	-	-	-		
2	-	-	-	-		
3	-	-	-	-		

**Table 3:** Correlation of pain assessment after the first visit of regenerative endodontic treatment

			6 h	12 h	24 h	48 h	72 h	96 h	120 h	144 h	168 h
Spearman’s rho	6 h	*r*	1,000	,773**	,622**	,448**	,072	,266	,245	,291	,440**
		*p*	.	,000	,000	,003	,645	,085	,113	,062	,003
		*N*	43	43	43	43	43	43	43	42	43
	12 h	*r*		1,000	,723**	,528**	,373*	,485**	,307*	,443**	,434**
		*p*		.	,000	,000	,014	,001	,045	,003	,004
		*N*		43	43	43	43	43	43	42	43
	24 h	*r*			1,000	,780**	,419**	,575**	,418**	,465**	,543**
		*p*			.	,000	,005	,000	,005	,002	,000
		*N*			43	43	43	43	43	42	43
	48 h	*r*				1,000	,430**	,485**	,353*	,335*	,533**
		*p*				.	,004	,001	,020	,030	,000
		*N*				43	43	43	43	42	43
	72 h	*r*					1,000	,736**	,641**	,509**	,546**
		*p*					.	,000	,000	,001	,000
		*N*					43	43	43	42	43
	96 h	*r*						1,000	,733**	,661**	,727**
		*p*						.	,000	,000	,000
		*N*						43	43	42	43
	120 h	*r*							1,000	,679**	,469**
		*p*							.	,000	,002
		*N*							43	42	43
	144 h	*r*								1,000	,577**
		*p*								.	,000
		*N*								42	42
	168 h	*r*									1,000
		*p*									.
		*N*									43

**Table 4:** Correlation of the consumption of analgesic tablets after regenerative endodontic treatment first visit

			6 h	12 h	24 h	48 h	72 h	96 h	120 h	144 h	168 h	
Spearman’s rho	6 h	*r*	1,000	,625**	,680**	,492**	,306*	,149	,309*	,309*	.	
		*p*	.	,000	,000	,001	,046	,339	,044	,044	.	
		*N*	43	43	43	43	43	43	43	43	43	
	12 h	*r*		1,000	,738**	,521**	,349*	,483**	,337*	,337*	.	
		*p*		.	,000	,000	,022	,001	,027	,027	.	
		*N*		43	43	43	43	43	43	43	43	
	24 h	*r*			1,000	,715**	,464**	,259	,424**	,424**	.	
		*p*			.	,000	,002	,094	,005	,005	.	
		*N*			43	43	43	43	43	43	43	
	48 h	*r*				1,000	,767**	,296	,470**	,470**	.	
		*p*				.	,000	,054	,001	,001	.	
		*N*				43	43	43	43	43	43	
	72 h	*r*					1,000	,373*	,563**	,563**	.	
		*p*					.	,014	,000	,000	.	
		*N*					43	43	43	43	43	
	96 h	*r*						1,000	,699**	,699**	.	
		*p*						.	,000	,000	.	
		*N*						43	43	43	43	
	120 h	*r*							1,000	1,000**	.	
		*p*							.	.	.	
		*N*							43	43	43	
	144 h	*r*								1,000	.	
		*p*								.	.	
		*N*								43	43	
	168 h	*r*									.	
		*p*									.	
		*N*									43	

### Correlations in pain assessment

A significant positive correlation was identified between pain levels at sequential time intervals. Specifically:

- The 6th and 12th hours, the 12th and 24th hours, the 24th and 48th hours, and the 72nd and 96th hours all showed highly significant positive correlations (*p* < 0.05).
- Pain assessments at the 96th, 120th, and 168th hours were also positively correlated (*p* < 0.05) **(**Table 3**).**

### Correlations in analgesic consumption

A significant positive correlation was observed in analgesic tablet consumption between the 12th and 24th hours, the 24th and 48th hours, and the 48th and 72nd hours (*p* < 0.05) (Table 4).

## Discussion

Clinical studies using regenerative endodontic procedures have shown high survival and success rates ranging from 95 to 100% [30, 31]. Estefan et al. [32] applied RET and found that more root development and successful treatment results were obtained in patients in the 9–13 age group compared to the 14–18 age group. This study included patients born between 2009 and 2015 for postoperative pain assessment.

The degree of postoperative pain varies depending on multiple physical and psychological factors related to the patient [33]. Additionally, it is difficult to determine whether irrigation solutions cause pain [34]. Other variables besides the irrigation protocol may also affect the level of postoperative pain. Those variables may be the type of tooth, degree of preoperative pain, and the size of the apical foramen [34, 35]. In the current study, the type and location of the teeth that are treated, the degree of preoperative pain, and the tooth’s developmental stage were standardized to minimize the effect of these variables on postoperative pain.

The success of RET depends mainly on the irrigation protocol. Chemical debridement not only provides intracanal disinfection but also has an inducing effect on the proliferation of stem cells and growth factors in the dentinal tubules [29]. EDTA has been widely recommended as a final irrigating solution [36]. The use of EDTA as an irrigant during REPs is essential as it promotes increased expression of factors associated with the differentiation of stem cells and, therefore, plays a vital role in the formation of new pulp tissues [29]. Additionally, it should be used together with NaOCl to minimize the cytotoxic effect caused by NaOCl and eliminate the smear layer [9]. However, EDTA has been reported to have limited ability to remove the smear layer in the apical third of the root compared to HEDP [37]. Failure to adequately clean the apical third of the root may lead to microleakage in the canal filling and failure in treatment.

For this reason, research is being carried out to develop alternative chelator agents with a robust decalcifying effect when used with NaOCl. Citric acid has been reported to be superior to the gold standard EDTA in stem cell release, attachment, and proliferation [38]. A statistically significantly greater TGF-β1 extraction was observed in the 10% citric acid group compared to EDTA [13], while another study also reported that cell viability of apical papilla stem cells was not affected by 10% citric acid irrigation [9]. Additionally, 10% citric acid was more effective than 17% EDTA in removing the smear layer [12]. When used with NaOCl, EDTA, and citric acid causes an almost complete loss of available chlorine. This reduces the effectiveness of NaOCl. In a study conducted by Zehnder et al. [36], it was reported that EDTA and citric acid negatively affected the effectiveness of NaOCl, while HEDP did not prevent the release of Cl, and their combined use may be more effective. In studies evaluating the effectiveness of EDTA and etidronic acid on stem cells and growth factors, it was observed that EDTA and HEDP had similar effects on growth factor release [39] and migration of stem cells [40]. Furthermore, compared with EDTA, HEDP is a less destructive agent in terms of its effects on root dentine. HEDP-NaOCl application in continuous chelation throughout biomechanical preparation inhibits smear layer formation while exhibiting minimal toxicity [41]. A recent in vitro study by Deniz et al. [40], demonstrated that 9% HEDP induced a greater release of TGF-b1 compared to 17% EDTA. However, the study also found that stem cell proliferation and viability in the apical papilla were higher EDTA than 9% HEDP. This in vivo current study compared the postoperative pain and analgesic use of two dual-action irrigating solutions for continuous chelation (HEDP and NaOCl) with dual-step NaOCl and EDTA irrigation.

NaOCl is the most commonly used root canal irrigant [42]. The use of NaOCl in high concentrations increases its antimicrobial effect but increases undesirable effects on dentin tissue [4]. The ability of 2.5% and 5.25% NaOCl to inhibit resistant bacterial cells in mature and old biofilms is equivalent to the effectiveness of 1,5% NaOCl [43]. Furthermore, no significant differences in pain and periapical healing were reported between the 1% and 5% NaOCl groups [44]. Irrigation was performed with 1,5% NaOCl in the current study.

Side-vented needles cause less postoperative pain compared to end-vented needles [45]. In addition, since irrigation solutions have a high risk of extrusion in teeth with open apexes, it is crucial to use side-vented needles. Therefore, in this study, irrigation was performed using a side-vented needle placed 1 mm shorter than the working length.

The ability of Ca-Si-based materials to penetrate dentinal tubules plays a crucial role in preventing bacterial regrowth within the tubules and enhancing the bond between the material and dentinal tissue [46]. Recent studies have investigated the impact of various final irrigation solutions and agitation techniques on the dentinal tubule penetration of premixed Ca-Si materials [46, 47]. These investigations have demonstrated that the penetration area of Ca-Si materials is significantly influenced by both the choice of root canal filling material and the irrigation protocol. Furthermore, findings suggest that the application of specific irrigation solutions, combined with agitation methods such as ultrasonic or sonic activation, can substantially improve the tubule penetration of Ca-Si materials. This enhanced penetration may contribute to better sealing ability, improved antibacterial efficacy, and superior biological outcomes, highlighting the importance of optimizing irrigation protocols in conjunction with the use of advanced premixed Ca-Si sealers.

In RET of immature permanent teeth with infected necrotic pulp, calcium hydroxide paste [48] or triple antibiotic paste (minocycline, ciprofloxacin, metronidazole) [49] has been recommended as an intracanal medication to eliminate all bacterial species in infected root canals. However, numerous studies have shown that high concentrations of antibiotics inhibit apical stem cell survival [50, 51]. The European Association of Endodontology recommends that their use be avoided due to the lack of solid evidence to support the use of antibiotics in regenerative endodontic procedures [52]. The attachment of apical papilla stem cells to root dentin is greater in calcium hydroxide than in triple antibiotic paste. Additionally, water-based calcium hydroxide increased the amount of (TGF)-β1 release compared to EDTA alone [53]. In the current study, calcium hydroxide paste was used as an intracanal medicament in addition to root canal irrigation to eliminate intracanal microorganisms.

This study used passive ultrasonic irrigation (PUI) to activate irrigation solutions because it has proven to be more effective in removing pulp tissue and eliminating the smear layer than traditional syringe irrigation [54]. Additionally, it has been reported to show superior effects compared to other dynamic activation systems [55]. Although PUI use may be associated with apical extrusion of debris, which should be avoided for regenerative endodontic applications, several studies have shown that with appropriate penetration length, extrusion may not pose a problem [55, 56]. In this study, activation was made with the UltraX device, which was 2 mm shorter than the working length, to prevent the extrusion of debris and irrigation solutions while performing PUI.

A limitation of this study is that the distance of MTA to the cementum-enamel junction may vary. The thickness of the MTA barrier, which was 3–4 mm in some teeth, was slightly more or less in others.

In the present study, pain assessment performed at different hours revealed that patients who used HEDP and NaOCl solution together had higher pain levels than other patients. HEDP was identified to exhibit short-term compatibility with NaOCl solutions at clinical strength, thus retaining the desired antimicrobial and proteolytic effects of NaOCl whilst adding an element of decalcification to the mixture [57]. In a survey by Tartari et al. [58], the impact of HEDP on periapical debris extrusion within the root canal was evaluated. The group in which a mixture of NaOCl and HEDP was used as an irrigation solution showed higher debris extrusion levels than sequential irrigation of EDTA and NaOCl. Researchers attributed this to the synergistic effect of NaOCl and HEDP [58]. Debris extrusion often leads to negative consequences such as flare-ups between appointments, postoperative pain, and delayed healing [59]. The high amount of debris extrusion observed when using a NaOCl mixture with HEDP could raise concerns during endodontic procedures [58]. However, a recent randomized clinical trial evaluating the inflammatory effects on periapical tissues of using a 2.5% NaOCl solution and a mixture solution of 2.5% NaOCl and 9% HEDP found no significant difference in patients’ postoperative pain levels after 24 h. This study stated that HEDP did not affect the clinical effects of NaOCl [60].

According to the findings obtained in the current study, the patient groups in which HEDP and NaOCl mixture solution was applied showed higher pain levels than those in which EDTA and citric acid solution were used. Additionally, higher pain levels were reported up to the 24th postoperative hour, especially in the patient group treated with HEDP and NaOCl mixture solution. This may be attributed to the high irritant effect of the NaOCl and HEDP mixture solution in the periapical region within 24 h postoperatively. In this study, postoperative pain levels differ in the pairwise comparison of citric acid and EDTA with HEDP - NaOCl mixed solution. Therefore, the first null hypothesis of the study was rejected.

In the present study, the incidence of analgesic intake has also been assessed as a secondary outcome. The frequency of analgesics taken by patients decreased over time in each tested group. There was no significant difference between the groups. Analgesic consumption decreased after 24 h for all groups. The second null hypothesis was accepted according to the current study’s results.

## Conclusions

Within limitations, the mixed solution of HEDP and NaOCl showed higher degrees of postoperative pain than EDTA and citric acid solution. However, these three groups obtained statistically comparable results in analgesic usage. These findings highlight the importance of selecting appropriate chelator solutions in regenerative endodontic procedures to minimize patient discomfort while ensuring effective treatment outcomes. Clinically, the results suggest that citric acid and EDTA may be preferable for reducing postoperative pain without compromising efficacy. Finally, this study emphasizes the need for further research to explore alternative chelator combinations that might enhance therapeutic outcomes while minimizing adverse effects. Future randomized clinical studies should also focus on assessing long-term consequences, such as apical closure, vitality status, and periapical healing, to provide a more comprehensive understanding of the implications of different irrigation protocols in regenerative endodontics.

## Data Availability

No datasets were generated or analysed during the current study.
